# Assessment of Fecundity and Germ Line Transmission in Two Transgenic Pig Lines Produced by Sleeping Beauty Transposition

**DOI:** 10.3390/genes3040615

**Published:** 2012-10-12

**Authors:** Wiebke Garrels, Stephanie Holler, Nicole Cleve, Heiner Niemann, Zoltan Ivics, Wilfried A. Kues

**Affiliations:** 1 Friedrich-Loeffler-Institut, Institute of Farm Animal Genetics, Höltystraße 10, 31535 Neustadt, Germany; E-Mails: wiebke.garrels@fli.bund.de (W.G.); stephanie.holler@fli.bund.de (S.H.); nicole.cleve@fli.bund.de (N.C.); heiner.niemann@fli.bund.de (H.N.); 2 Paul-Ehrlich-Institute, Paul-Ehrlich-Straße 51-59, 63225 Langen, Germany; E-Mail: zoltan.ivics@pei.de

**Keywords:** transgenic animal, germ line transmission, active transgenesis, hyperactive transposase, humanized pig model, livestock, cytoplasmic plasmid injection, gene silencing, permissive locus

## Abstract

Recently, we described a simplified injection method for producing transgenic pigs using a non-autonomous Sleeping Beauty transposon system. The founder animals showed ubiquitous expression of the Venus fluorophore in almost all cell types. To assess, whether expression of the reporter fluorophore affects animal welfare or fecundity, we analyzed reproductive parameters of two founder boars, germ line transmission, and organ and cell specific transgene expression in animals of the F1 and F2 generation. Molecular analysis of ejaculated sperm cells suggested three monomeric integrations of the Venus transposon in both founders. To test germ line transmission of the three monomeric transposon integrations, wild-type sows were artificially inseminated. The offspring were nursed to sexual maturity and hemizygous lines were established. A clear segregation of the monomeric transposons following the Mendelian rules was observed in the F1 and F2 offspring. Apparently, almost all somatic cells, as well as oocytes and spermatozoa, expressed the Venus fluorophore at cell-type specific levels. No detrimental effects of Venus expression on animal health or fecundity were found. Importantly, all hemizygous lines expressed the fluorophore in comparable levels, and no case of transgene silencing or variegated expression was found after germ line transmission, suggesting that the insertions occurred at transcriptionally permissive loci. The results show that Sleeping Beauty transposase-catalyzed transposition is a promising approach for stable genetic modification of the pig genome.

## 1. Introduction

Transgenic animals are important disease models for biomedical research [1,2,3,4,5]. In laboratory mice the most commonly used techniques for transgenesis are pronuclear injection of foreign DNA into fertilized oocytes, and embryonic stem cell-mediated gene targeting [6,7]. With the ongoing genome projects of domestic species and the advances in genetic engineering, it is anticipated that genetically modified large mammals will increasingly complement the commonly used small animal models in biomedicine and pharmaceutical research, since several aspects of human diseases such as progression, physiology, metabolism and aging are not properly mirrored in small animal models [8,9]. At the moment, the generation of transgenic livestock is an expensive and inefficient process, mainly because of the lack of authentic embryonic stem cells in these species [10,11]. The first established method for stable transgenesis in livestock was the pronuclear injection of foreign DNA into a pronucleus of a zygote. The high content of lipids in porcine and bovine zygotes required high speed centrifugation to visualize the otherwise hidden pronuclei; with potentially compromising effects on their developmental competence [12]. During recent years alternative methods have been assessed, including sperm-mediated DNA transfer [13,14,15], injection of oocytes with retro- and lentiviruses [16,17] and somatic cell nuclear transfer (SCNT) [18,19,20]. The main problem associated with these techniques is, that only a small fraction of transgenic offspring show the expected phenotype, because the foreign DNA is “passively” integrated into the genome [21,22,23]. The term passive integration highlights that the site of integration cannot be controlled, but depends on sites of DNA double strand breaks randomly induced by physical or chemical mutagens [21,22,23]. It appears that these passive sites often do not permit faithful transgene expression following germ line transmission.

In contrast, recent developments of highly specific (exogenous) DNA modifying enzymes allow the performance of precise genetic modifications and to preferentially target transcriptional permissive genomic loci by employing non-viral transposase or integrase approaches [24,25,26,27,28,29,30,31]. In theory, the preference of DNA integrations into permissive loci may affect livestock welfare, and concerns due to potential insertional mutagenesis and effects of transgenesis in general have been raised [32,33,34,35]. Indeed only few studies have performed detailed health assessment of transgenic livestock [36,37] and provided long term results [36,37,38]. Pathomorphological phenotypes have been shown in transgenic swine and cattle derived from microinjected zygotes or reconstructed embryos derived by somatic cloning [34,36,39,40,41,42,43,44]. Long term studies on germ line transmission and general reproductive parameters have been followed in cattle, carrying a lentiviral phosphoglycerate kinase (PGK) promoter driving an enhanced green fluorescent protein (EGFP) [38], and in lentivirus transgenic pigs with systemic expression of EGFP [37].

Recently, we produced transgenic pigs with a hyperactive Sleeping Beauty (SB) transposon system [25], which represents one example for active transgenesis by an exogenously delivered transposase enzyme [21]. Founder animals showed ubiquitous expression of the Venus reporter transposed into the porcine genome.

**Figure 1:**
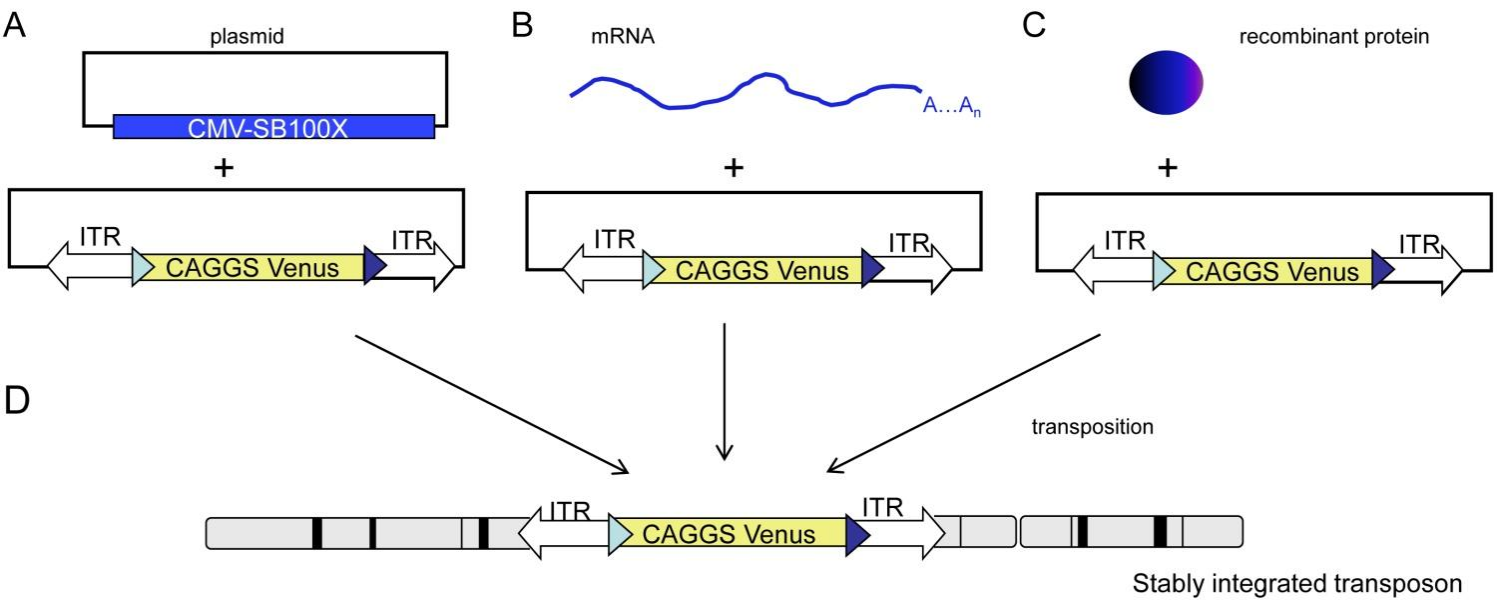
Potential combinations of Sleeping Beauty components. (A) Cytoplasmic injection of an expression plasmid encoding Sleeping Beauty transposase and a plasmid carrying a SB-transposon; (B) Cytoplasmic injection of Sleeping Beauty-mRNA and a SB-transposon; (C) Injection of recombinant Sleeping Beauty protein and a SB-transposon; (D) In all possibilities stable transposition into a chromosome occurs, however, it is likely that A-C differ in the kinetics and potential remobilization events. CMV-SB100x, cytomegalovirus immediate early promoter driving a SB100x-cDNA; CAGGS, cytomegalovirus enhancer, chicken beta actin hybrid promoter driving a Venus-cDNA; ITR, SB specific-inverted terminal repeat; blue triangles, heterospecific loxP sites for a potential recombinase-mediated cassette exchange [25].

Integration of a transposon system occurs in two main steps: (i) precise excision of the transposon cassette from a donor plasmid at the ends of the flanking recognition sequences (inverted terminal repeats), and (ii) integration into the genome at a TA dinucleotide. In principle, the transposon plasmid can be supplemented with the required transposase activity by (i) a helper plasmid carrying an expression cassette of SB, (ii) in vitro-synthesized SB-transposase-mRNA or (iii) recombinant SB-transposase protein (Figure 1). The potential combinations will likely vary with respect to kinetics of integration, stability of the components and potential remobilization of primary integration events. After metabolization of the SB transposase, the integrated transposon will be fixed at a defined position in the genome. 

Here, we assessed health and fecundity of two founder boars, both carrying three monomeric integrations of the transposon and expressing high levels of the transgene. This is the first assessment of animal welfare and reproductive aspects of transgenic large mammals generated by an active transgenesis approach with a hyperactive transposase.

## 2. Results and Discussion

### 2.1. Generation and Analysis of Founder Animals

**Figure 2:**
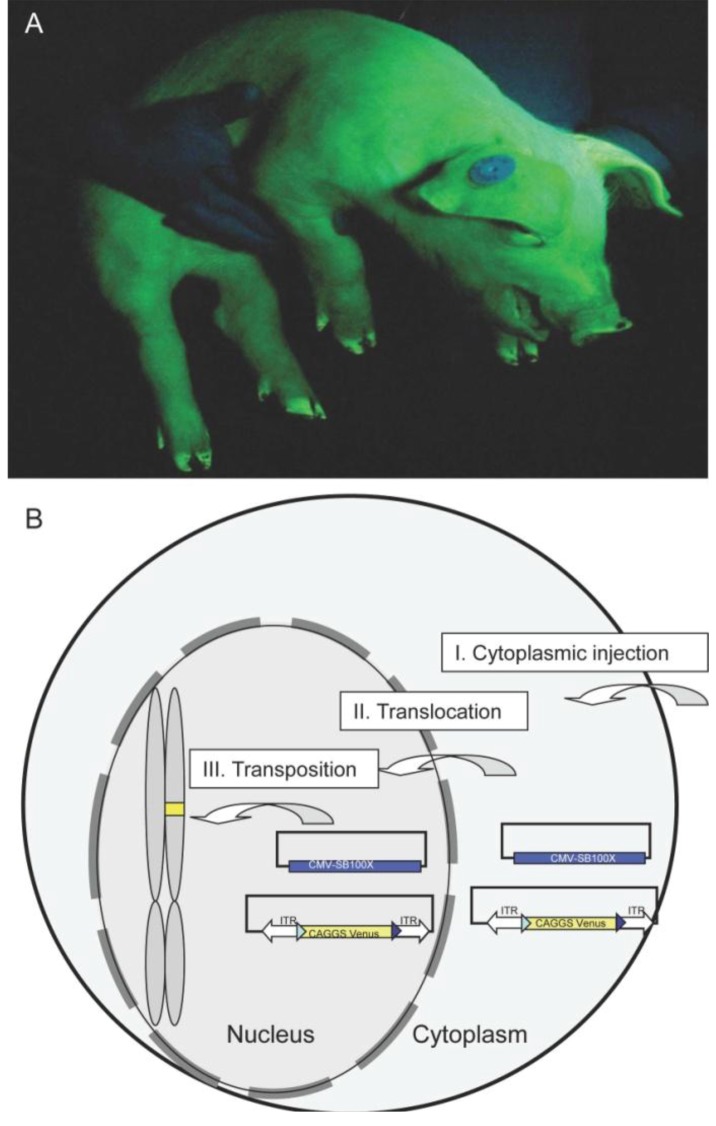
Transposon transgenic pig expressing Venus reporter. (A) Boar #505 under specific Venus excitation at the age of four weeks, Venus fluorescence is indicated by green color. Note the blue appearance of the hands holding the pig is due to reflected and scattered excitation light; (B) Scheme of CPI injection. After injection of the plasmid solution into the cytoplasm (I.), the plasmids translocate into the nucleus (II.). Expression of the SB100× helper plasmid results in the production of active SB protein and specific transposition of the transposon (without plasmid backbone) into the genome (III.).

The founder animals were produced with a simplified DNA injection method of circular DNA plasmids into the cytoplasm of porcine zygotes [25,45]. We presume that the CPI techniques result in higher developmental rates of porcine zygotes, because the fractionation of cellular components by high speed centrifugation is omitted. To produce the transgenic founders, we combined the non-autonomous hyperactive Sleeping Beauty (SB) 100x-system [46], with CPI [45]. The components of a non-autonomous transposon system were separated on two different expression plasmids. The injected zygotes were surgically transferred to synchronized foster sows. The first litter was delivered at term (day 114) and had a size of 12 piglets, of which five were transgenic. All piglets had a normal birth weight (750–1,250 gram), were healthy and had normal behavior. A normal sex ratio was found, and no tendon problems or other malformations were detected [47,48]. Two Venus-transgenic boars (#503 and #505) were raised to sexual maturity. Figure 2 shows one of the F0 boars at the age of one month under specific Venus excitation. Southern blot analysis indicated that the transgenic founder boars carried three monomeric transposons [25]. Previously, we sequenced a total of 25 integration sites of the Venus transposon [25], however due to gaps and ambiguities in the current pig genome data, only five could be assigned to porcine chromosomes X, 3, 7, 8 and 13. Importantly, no random integrations of plasmid backbone sequences or of the SB transposase helper plasmid were found [25]. Here, we present a detailed report of reproductive parameters and germ line transmission of the founder boars, and cellular and molecular analyses of their offspring. 

### 2.2. Analysis of Spermatozoa from Founder Boars

**Figure 3:**
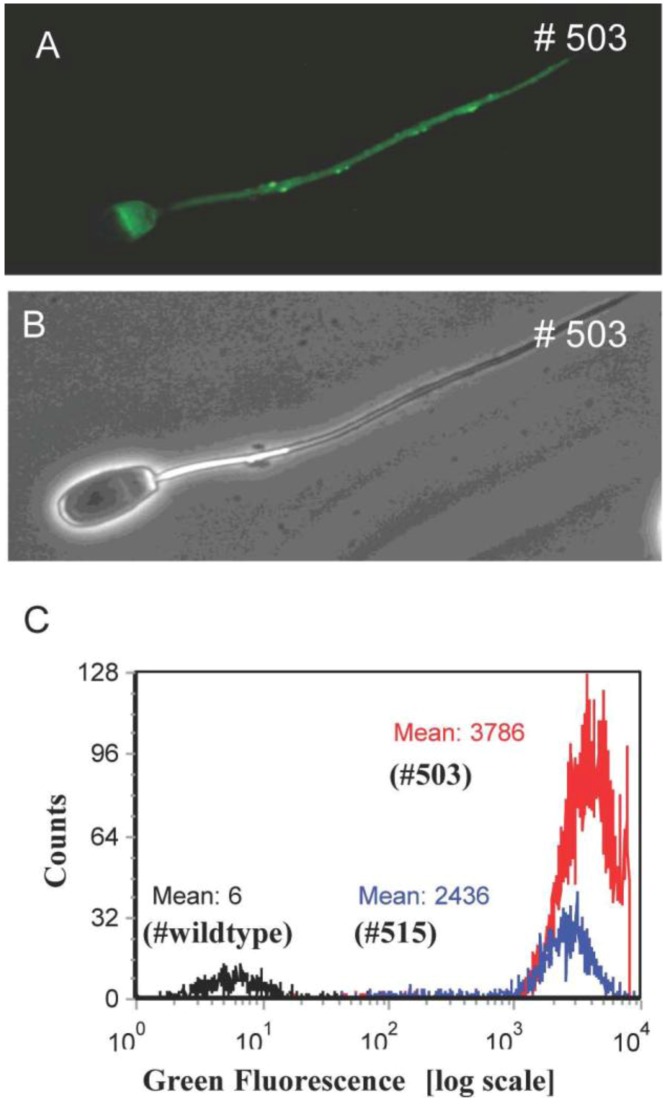
Expression of Venus fluorescence in spermatozoa. (A) Specific Venus fluorescence of a spermatozoon from F0 boar #503; (B) Brightfield image of A; (C) Flow cytometric analysis of pig spermatozoa. The black line in the FACS analysis represents wild type sperm; blue line, spermatozoa from F1 boar with single integration; red line, spermatozoa from boar #503 with three integrations. Note that all spermatozoa from the transgenic pigs are uniformly Venus-positive.

At the age of eight months, the founder boars (#503 and #505) were trained to use a dummy, both boars showed a good libido and ejaculated sperm could be recovered. Interestingly, all spermatozoa were uniformly Venus-positive. According to Mendelian rules, segregation of the three monomeric transposons should result in 12.5% triple-, 37.5% double-, 37.5% mono- and 12.5% non-transgenic sperm cells [49]. Apparently, the Venus protein was uniformly distributed between the developing sperm cells, which are connected via cytoplasmic bridges. This equal distribution of cytoplasmic components during spermatogenesis did not correlate with the genotype (Figure 3). A detailed analysis of ejaculated spermatozoa from the founder boar is given in reference [49]. Assessment of general parameters of sperm fertility showed that ejaculates from the transgenic boars fulfilled standard semen quality requirements, which included motility, velocity and morphological aspects [49].

### 2.3. Assessment of Germ Line Transmission and Derivation of F1-lines

To test the fertility of transgenic sperm, wild type sows were artificially inseminated with semen from the two Venus transgenic boars (#503 and #505), respectively. Six wild type sows were inseminated at natural heat and five became pregnant. Subsequently, three transgenic F1 sows were inseminated with founder semen; all three became pregnant and delivered healthy piglets at term. In total, 62 piglets were born, of which 81% were transgenic (Table 1). Molecular analysis confirmed segregation of the monomeric transposons in the offspring (see below). The piglets showed a normal birth weight, normal growth and social behaviour. The transgene expression did not affect health of the piglets. Previously, the most common clinical signs of disease associated with transgene expression in pigs included lethargy, lameness, uncoordinated gait, exophthalmos, and thickened skin [50]. Most likely, this phenotype was caused by ectopic expression of recombinant growth hormone. EGFP-transgenic piglets, produced with a lentiviral vector, were similar to wildtype animals with regard to reproductive parameters, behavior and general welfare criteria [37]. Here we extend these observations to transposon transgenic pigs, which showed an ubiquitous expression of the Venus fluorophore. Venus is an improved version of the yellow fluorescent protein (YFP) with stronger relative fluorescence, improved refolding after denaturation, and an increased pH resistance [51]. Compared to EGFP, the Venus protein carries several amino acids exchanges.

**Table 1:** Germ line transmission of transposon transgenic pigs and sex ratio of the offspring.

Breeding of boar / sow	Pregnancies after artificial insemination	Litter number	Born piglets	Ratio female / male	Transposon positive (%)	Ratio female / male of transposon positive piglets	Ratio female / male of non-transgenic piglets
F0 #503 / wt	3 / 3	1	8	5 / 3	5 (63)	3 / 2	2 / 1
		2	13	3 / 10	12 ( 92)	3 / 9	0 / 1
		3	9	4 / 5	7 (78)	3 / 4	1 / 1
F0 #505 / wt	2 / 3	4	6	1 / 5	5 (83)	1 / 4	0 / 1
		5	8	2 / 6	6 (75)	2 / 4	0 / 2
*Subtotal*	*5*		*x = 8.8, n = 44*	*15 / 29*	*35 (80)*	*12 / 23*	*3 / 6*
F0#505 /	1/1	6	10	3 / 7	8 (80)	2 / 6	1 / 1
F1#518							
F0#503 /	2/2	7	3	2 / 1	2 (67)	2 / 0	0 / 1
F1#537		8	5	3 / 2	5 (100)	3 / 2	0 / 0
F1#538							
*Subtotal*	*3*		*x = 6.0, n = 18*	*8 / 10*	*15 (83)*	*7 / 8*	*1 / 2*
Total	8		x = 7.8, n = 62	23 / 39	50 (81)	19 / 31	4 / 8

**Figure 4:**
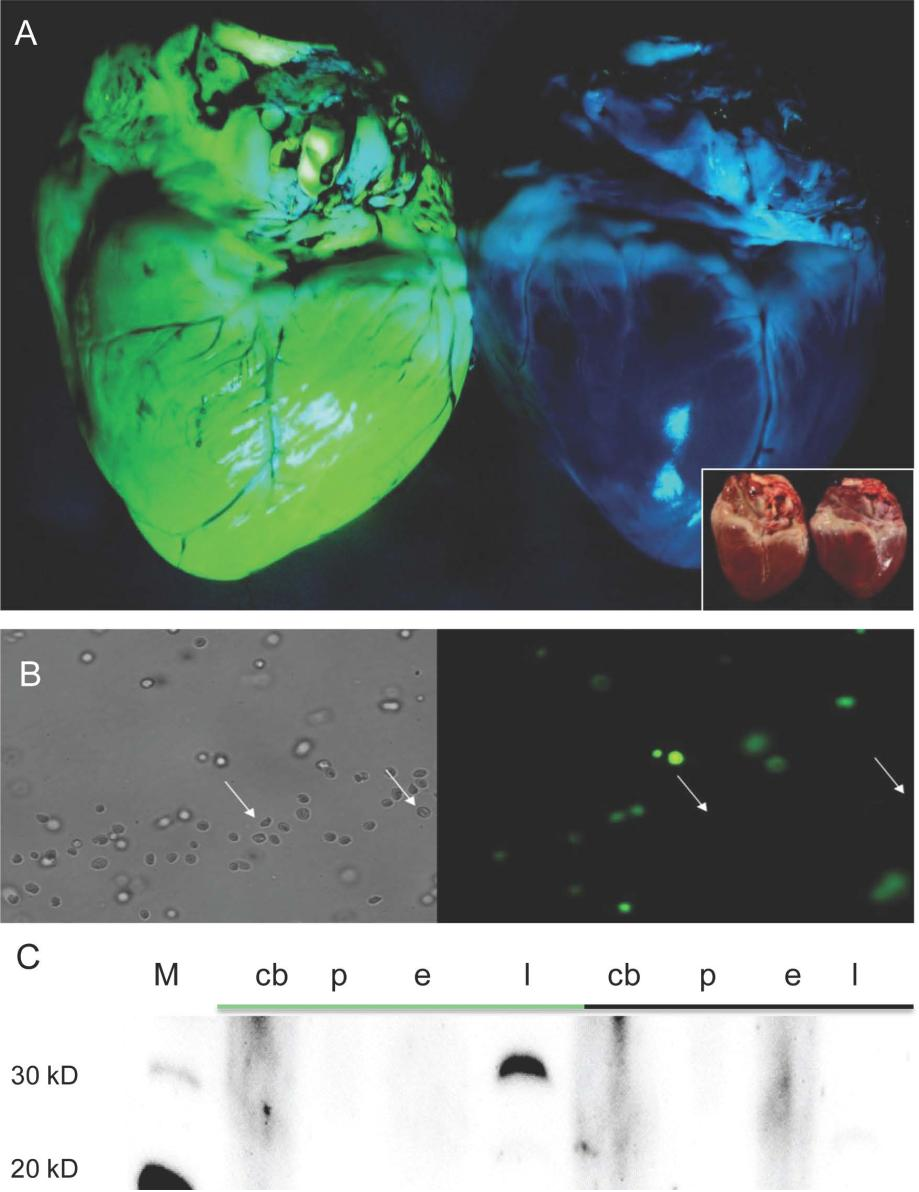
Expression of Venus fluorophore in the pig heart and absence of Venus in erythrocytes. (A) Exemplarily, a transgenic (left) and a wild type (right) pig heart are shown under specific excitation of the Venus fluorophore. Inset, brightfield view; (B) Smear of blood cells. Brightfield view (left), specific Venus excitation (right). Note, red blood cells showed no Venus expression (some erythrocytes are indicated by an arrow); (C) Western blot detection of Venus in blood fractions. In blood, the Venus expression is restricted to leucocytes. Green bar indicates samples from Venus transgenic pig; black bar samples from wild type pig. M, molecular size marker; cb, complete blood; p, plasma; e, erythrocyte fraction; l, leucocyte fraction. Apparent molecular weight of Venus protein is ~30 kD.

A direct correlation between the copy number of monomeric transposons and the fluorescence intensity in organs and cells was evident. Expression of the Venus reporter was observed in all organs (Figure 4) and almost all cell types of F1-animals. Only erythrocytes were found to be Venus-negative by fluorescence microscopy (Figure 4). To exclude the possibility that the specific fluorescence properties of Venus were quenched by haemoglobin or other red blood cell components, blood samples of F1-animals were fractionated in plasma, leucocytes and erythrocytes and used for immunoblotting. Western blot analyses revealed Venus protein in leucocytes, but not in erythrocytes. Thus the CAGGS-promoter did not seem to be active in porcine erythrocytes or their precursor cells (Figure 4). Albeit mammalian erythrocytes lack a nucleus, their precursor cells do have a nucleus and all proteins necessary during the lifespan of an erythrocyte have to be synthesized prior to degradation of the nucleus. Previously, we showed that functional Venus protein was also deposited in “extracellular” hair of these pigs, and that its fluorescent properties remained stable over periods of months [52].

**Figure 5:**
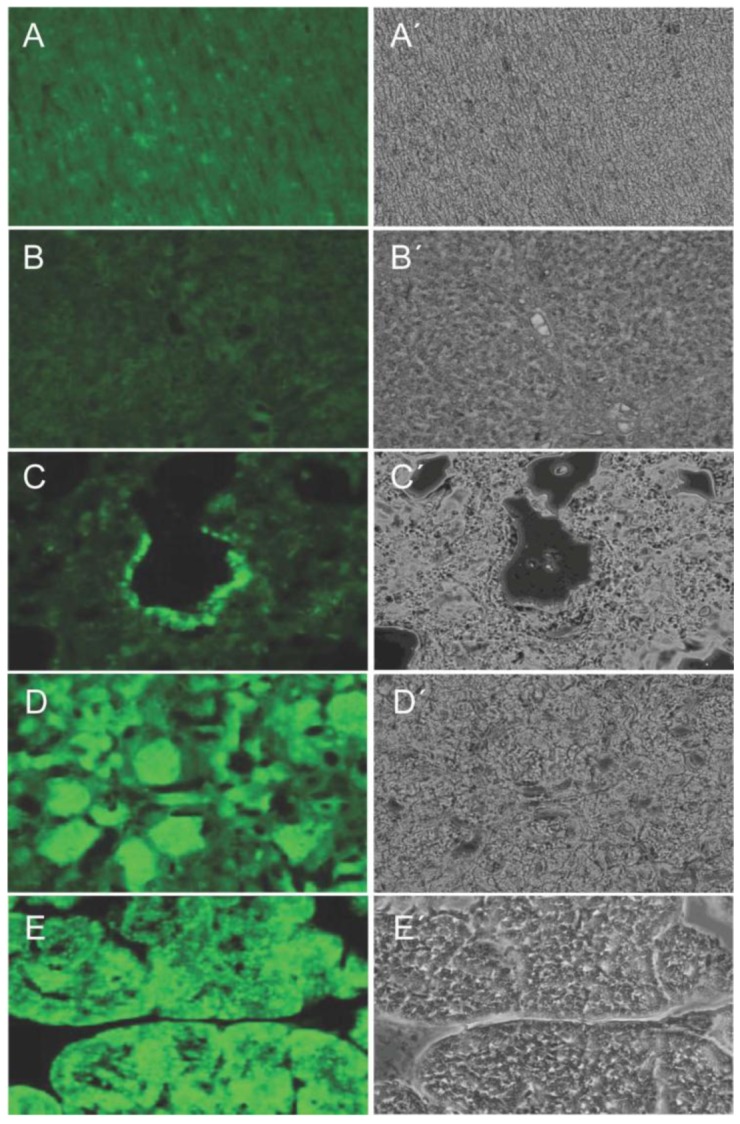
Organ and cell type-specific expression of Venus. Cryosections (10 µm) of A. heart, B. liver, C. lung, D. kidney, and E. pancreas of a Venus transgenic F1 pig are shown under normalized fluorescence excitation (left) and brightfield conditions (right). Almost all cells express the Venus reporter, however at cell type-specific levels.

A histological examination showed expression in all solid organs of transgenic F1 animals containing different transposon integrations, and all cells expressed the transgene (Figure 4, Figure 5). The level of expression varied between cell types, but specific cell types showed consistent expression levels. High expression levels were detected in kidney and pancreas, whereas heart, lung and liver showed moderate to low expression (Figure 5).

No case of variegated or silenced expression was found, whereas in previous studies of transgenic pig lines derived by random transgene integration, transgene silencing was a common finding [36,53].

**Figure 6:**
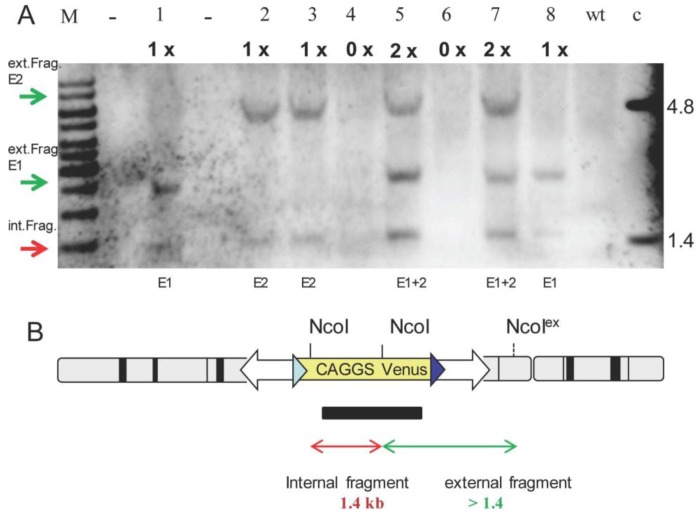
Southern blot analysis of F1 offspring. (A) Southern blot of eight F1 offspring (litter 5, Tab 1). 1×, 2× and 0× indicate the segregated transposon copy numbers. Piglets #1, #2, #3, #8 (lanes 1, 2, 3, 8) carry one copy of a monomeric transposon, piglets #5 and #7 carry two transposon copies, and #4 and #6 are non-transgenic. Red arrow: internal fragment ~1.4 kb. Green arrows: external fragments of the first and second integrant. Note: In this litter was no piglet with three transposon copies. M, marker; 1-12, lane number; - no sample was loaded; wt, wild type DNA; c, positive control; (B) Design of Southern blot. Genomic DNA was digested with NcoI, which releases an internal fragment of 1.4 kb and an external fragment of >1.4 kb depending on neighboring sequences of the transposon. The black bar indicates position of Digoxigenine labeled probe, which hybridizes to internal and external fragments. Thus each integrated transposon should result in a 1.4 and a > 1.4 kg fragment. Illegitimate integration can be excluded by employing restrictions flanking the ITR on original transposon plasmid, for details see [25].

Ubiquitous expression of Venus was confirmed by Northern blotting and Western blot detection of Venus protein [25,49]. Southern blot genotyping confirmed segregation of the monomeric transposons and supported the identification of F1 animals carrying identical integration events of the transposon (Figure 6). Thus the two founders (each with three monomeric transposons) can be used to derive six independent transgenic pig lines with a monomeric transposon. Germ line transmission of the SB transposons was not affected by the integrations site. In the absence of exogenously supplied Sleeping Beauty transposase, no changes in the integration sites (remobilisation) were found, consistent with the lack of endogenous SB-like transposons in the porcine genome. 

### 2.4. Crossbreeding to Generate Piglets with Maximized Transposon Copy Number and Venus Expression

**Figure 7:**
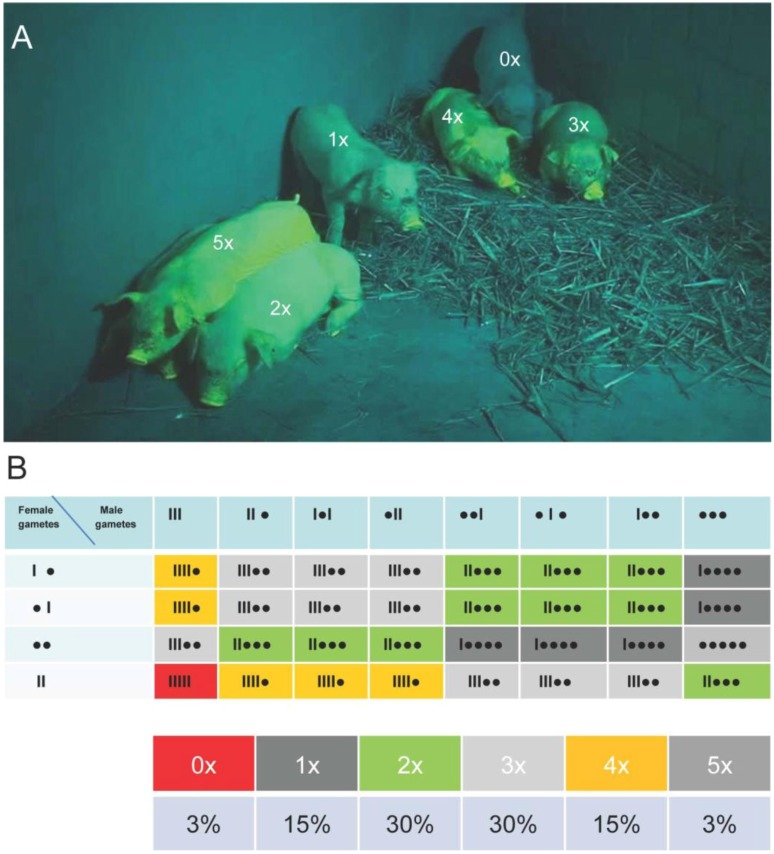
Maximized Venus expression by crossbreeding. (A) Female F1 sow # 518 (descendant of boar # 503) was inseminated with semen of the F0 boar #505. Seven of the F2 offspring (Table 1, litter 6) are shown under specific Venus excitation. The piglets are labeled with the copy number of the transposon cassette. The number of integration sites of the Venus transposon shows a direct correlation to fluorescence intensity; (B) Calculated transposon segregation for parents with three and two independent integrants of the Venus transposon. Dot, Venus transposon integrant; line, wt allele.

To assess, whether the Venus protein behaves “neutral” in transgenic pigs, or whether (detrimental) effects become obvious above a critical expression level, three F1 sows, carrying two transposon copies, were inseminated with sperm from founders #503 or #505 (Table 1). The sows delivered 18 piglets, which carried up to five monomeric transposons. Again, a direct correlation between transposon copy number of the carrier, and the specific fluorescence intensity was apparent (Figure 7). Detrimental effects of the Venus reporter with respect to vital parameters, such as birth weight, litter size and animal health were not found. Piglets, which carried five different Venus transposons at permissive loci, exhibited the highest fluorescence, and no critical expression level could be determined. Apparently breeding to F2 generation neither affected expression, nor animal health.

The mean littersize of 7.8 piglets for the germ line transmission experiments (Table 1) is smaller than the mean littersize of wild type pigs in the Institutes pig facility. However, this may be due to different selection criteria for these two populations of pigs. Whereas wild type animals are strongly selected for high fecundity, and animals which give rise to small litter sizes are slaughtered, the transposon founders were solely selected on the presence of the transgene. Also a higher ratio of male (62%) than female offspring (38%) was recorded (Table 1). This was found both in the transgenic and in the non-transgenic offspring of transposon transgenic parental animals, and did not correlate with the transgenic status. The smaller litter size and the altered sex ratio warrant further studies into the role of transposon transgenesis on porcine fertility.

### 2.5. Generation of Porcine Models for Biomedical and Pharmaceutical Research

Importantly, the active transposition in porcine zygotes did not require any antibiotic selection cassettes, which are un-wanted DNA elements according to guidelines provided by regulatory authorities like the European Medicines Agency (EMA) and the Food and Drug Administration (FDA) in the USA. Recent experimental data in transgenic mice and cattle support the notion that the presence of an antibiotic selection cassette may result in variegated expression of the gene of interest [28,54]. Thus gene transfer strategies, which omit or allow removal of unwanted “transgenic” DNA sequences, are necessary to fully exploit transgenic animal models.

Methodological improvements in gene transfer have led to a rapidly increasing list of biomedical livestock models during recent years [55,56,57,58,59,60,61,62]. Transgenic pigs have been shown to mimic genetic and metabolic human diseases [1,5,55,56,57,58,60,61,62,63]. An important example is the porcine cystic fibrosis model, which shows a similar disease phenotype as for human patients [64], whereas transgenic mouse models failed to exhibit lung, pancreatic and intestinal obstructions. Huntington´s disease is a neurodegenerative disorder characterized by expression of a mutated huntingtin. A transgenic pig model expressing the N-terminal huntingtin with a polyglutamine tract seems to accumulate misfolded proteins in neurons, and initiated apoptosis in the affected neurons [56]. Photoreceptor topography in the pig retina is highly similar to that in humans, as it includes a cone rich, macula-like area centralis. Transgenic pigs expressing macular dystrophy-causing mutation in the ELOVL4 (elongation of very long chain fatty acids-4) gene showed photoreceptor loss, disorganised inner and outer segments, and diminished electro-retinography responses, suggesting that the transgenic pigs mirror macular degeneration and provide a unique model for therapeutic interventions [65]. Recently, the first immune deficient pigs were cloned [66,67,68], which is promising for large animal models for cell transplantation experiments. Our results suggest that Sleeping Beauty transposon-catalyzed stable transgene delivery is a useful addition in the toolkit for improved genetic engineering of the porcine genome for the generation of novel disease models [69].

## 3. Experimental Section

### 3.1. Animals and Samples

Animals were maintained and handled according to German laws according animal welfare and genetically modified organisms (GMO). All animal experiments were approved by an independent ethics committee. Sperm rich fractions were collected using a dummy and by the gloved hand technique. Sperm samples were extended with Androhep (1:1). For artificial insemination, semen was diluted to a final concentration of 10^8^ sperm cells/mL. For genotyping, blood and ear biopsies were collected, and DNA was isolated with the proteinase K method. 

### 3.2. Southern Blotting

Southern blot was done according to standard protocols. The genomic DNA was digested with NcoI. Hybridization with a CAGGS-Venus probe resulted in a constant internal fragment of around 1.4 kb, and a variable external fragment of >1.4 kb per integration [25]. Southern blotting was done according to standard protocols. 

### 3.3. Sperm Analysis

Flow cytometry analysis of spermatozoa was performed using a FACScan (BD Bioscience) equipped with an argon laser (488 nm, 15 mW). Samples were diluted to 0.5 × 10^6^ cells/mL and measured in duplicates acquiring 10,000 cells per sample. 

### 3.4. Western Blotting

Cells were extracted in RIPA buffer and 10 microgram of protein per slot was separated on a SDS-PAGE gel, blotted to a PVDF membrane, blocked and probed with a rabbit polyclonal anti-EGFP antibody (Santa Cruz) in 1:1000 dilution, followed by a secondary anti-rabbit antibody in 1:10 000 dilution (Sigma-Aldrich). For detection an ECL+ kit (GE Healthcare) and an image acquisition system (Vilber Lourmat, Fusion SL 3500) were used. For complete Western blotting protocol see references [49,52].

### 3.5. Macroscopic Imaging and Fluorescence Microscopy

Venus transgenic and wildtype animals were excited with a blue LED and images were recorded with a digital camera and an emission filter (Lee Filter). Isolated organs were recorded in an identical manner. Organ samples were fixed in neutralized 4% formaldehyde overnight, then transferred to a 20% sucrose solution, and frozen in embedding medium (Micom Laborgeräte, Walldorf, Germany) on dry ice. This treatment excellently preserved the Venus fluorescence. In a cryotome, 10 μm sections were cut, mounted in Vectashield and viewed an an Olympus BX60 equipped with epifluorescence (excitation: 450–490 nm; emission: 515–550 nm; dichroic mirror: 505 nm). Normalized images were recorded with a scientific digital camera (Olympus DP71).

## 4. Conclusions

We have established the use of a Sleeping Beauty transposon system for the generation of germ line competent transgenic pigs by CPI. Generation of transgenic pigs with the transposon-system seems to be an efficient method for the production of large animal models and seems to avoid unwanted side effects of transgenesis, such as insertional mutagenesis, integration of concatemeres or integration into transcriptional non-permissive loci. In contrast to conventional random transgene integration, the transposase-catalyzed DNA integration preferentially resulted in monomeric integrations in transcriptionally permissive loci of the genome. Together with more accurate whole genome data and highly specific designed DNA modifying enzymes [59], precision genetic modifications became feasible in the pig genome [21,25,70]. It is anticipated that authentic pluripotent cells of the pig will be generated in the near future. Thus transgenesis in the pig will be significantly improved for the generation of humanized pig models [71]. The expected progress of pig transgenesis with increased success rates and decreased costs, will make the pig an attractive complementary model for advanced approaches in biomedical and pharmaceutical research.
